# A website for unhealthy alcohol use: how to make it visible and to whom?

**DOI:** 10.1186/1940-0640-7-S1-A51

**Published:** 2012-10-09

**Authors:** Nicolas Bertholet, Myriam Rege-Walther, Bernard Burnand, Jean-Bernard Daeppen

**Affiliations:** 1 Center for Alcohol Treatment, Department of Medicine and Public Health, Lausanne University, Lausanne, Switzerland; 2 Health Care Evaluation Unit, Lausanne University Hospital, Lausanne, Switzerland

## 

Websites providing information and tailored feedback for unhealthy alcohol use are increasingly used to reach a large population that does not necessarily access primary-care services. Such websites need to target individuals with unhealthy alcohol use and to be known of and visited to be efficacious. We developed a French-language website offering general information on alcohol use, screening, and brief intervention with tailored feedback. To increase the site’s visibility, we conducted a media campaign in the French part of Switzerland. We assessed the characteristics and satisfaction of web visitors using a screening questionnaire and satisfaction survey built into the site. To qualify the impact of the media campaign, we recorded the geographical provenance of the users. Between July 15, 2011 (the official site release date) and January 31, 2011, 15,633 unique visitors accessed the website, and 84% (13,160) completed the screening and received tailored feedback. General information pages represented 25% of the 28,986 visited pages. Most users were men (67%); the mean age was 36.3 years (standard deviation, 13.6 years). Thirty-four percent of men and 38% of women reported risky alcohol use (> 14 drinks per week for men, >7 for women), and 54% of men and 30% of women reported heavy episodic drinking (> 6 drinks in a single occasion) at least once a month. Of the 56% people with unhealthy alcohol use, 66% envisioned change after receiving the feedback. Among those who completed the satisfaction survey (n = 1001), 88% said the website provided useful information. Most visits (83%) came from Switzerland. People may visit websites providing information and tailored feedback on alcohol use on their own, but a media campaign appear to greatly increase the number of visitors. Our website targets the appropriate users, since unhealthy alcohol use was overrepresented among visitors compared with the general population, and satisfaction was high. Most at-risk drinkers envisioned change after their visit.

